# Hypoperfusion of the Adventitial Vasa Vasorum Develops an Abdominal Aortic Aneurysm

**DOI:** 10.1371/journal.pone.0134386

**Published:** 2015-08-26

**Authors:** Hiroki Tanaka, Nobuhiro Zaima, Takeshi Sasaki, Masaki Sano, Naoto Yamamoto, Takaaki Saito, Kazunori Inuzuka, Takahiro Hayasaka, Naoko Goto-Inoue, Yuki Sugiura, Kohji Sato, Hirona Kugo, Tatsuya Moriyama, Hiroyuki Konno, Mitsutoshi Setou, Naoki Unno

**Affiliations:** 1 Division of Vascular Surgery, Hamamatsu University School of Medicine, Hamamatsu, Japan; 2 Department of Cell Biology and Anatomy, Hamamatsu University School of Medicine, Hamamatsu, Japan; 3 Second Department of Surgery, Hamamatsu University School of Medicine, Hamamatsu, Japan; 4 Department of Anatomy and Neuroscience, Hamamatsu University School of Medicine, Hamamatsu, Japan; 5 Department of Applied Biological Chemistry, Graduate School of Agriculture, Kinki University, Nara, Japan; 6 Health Innovation & Technology Center, Hokkaido University, Sapporo, Japan; 7 Department of Health Promotion Science, Tokyo Metropolitan University, Tokyo, Japan; 8 Department of Biochemistry, School of Medicine, Keio University, Tokyo, Japan; Nagoya University, JAPAN

## Abstract

The aortic wall is perfused by the adventitial vasa vasorum (VV). Tissue hypoxia has previously been observed as a manifestation of enlarged abdominal aortic aneurysms (AAAs). We sought to determine whether hypoperfusion of the adventitial VV could develop AAAs. We created a novel animal model of adventitial VV hypoperfusion with a combination of a polyurethane catheter insertion and a suture ligation of the infrarenal abdominal aorta in rats. VV hypoperfusion caused tissue hypoxia and developed infrarenal AAA, which had similar morphological and pathological characteristics to human AAA. In human AAA tissue, the adventitial VV were stenotic in both small AAAs (30–49 mm in diameter) and in large AAAs (> 50 mm in diameter), with the sac tissue in these AAAs being ischemic and hypoxic. These results indicate that hypoperfusion of adventitial VV has critical effects on the development of infrarenal AAA.

## Introduction

An abdominal aortic aneurysm (AAA) is a common degenerative disease of the aorta that is characterized by impaired aortic wall integrity that can lead to dilatation and ultimately to fatal rupture; people aged > 65 years are most commonly affected [1]. Risk factors for the development of AAA include smoking, hypertension, male sex, and history of cerebrovascular or cardiovascular atherosclerosis [2]. Key features associated with AAA development include significant degeneration of the extracellular matrix induced by proteolytic enzymes, particularly matrix metalloproteinases (MMPs) [3], increased oxidative-stress, and chronic inflammation characterized by invasion of macrophages and mononuclear lymphocytes [4].

Measurements of tissue oxygen in the aortic wall during open repair of infrarenal AAAs have shown decreased levels of tissue oxygen [5]; the expression of hypoxia-inducible factor 1α (HIF-1α) has also been reported in human AAAs [6, 7]. A thick intraluminal thrombus (ILT) in the aneurysmal sac has been considered to prevent oxygen diffusion from the luminal blood flow[5]. Therefore, tissue hypoxia has been proposed as one of the factors exacerbating inflammation and aneurysmal development, consistent with the fact that MMP-2 is activated under hypoxic conditions by a signaling cascade beginning with HIF-1α [8]. Normally, oxygen is provided to the aortic tissue either through direct diffusion from aortic blood flow or from perfusion of the adventitial vasa vasorum (VV). Thus, we hypothesized that hypoperfusion of the VV may cause hypoxia in the aortic wall due to tissue ischemia. We have recently reported that the tissue in the aneurysmal sac becomes more ischemic than that in the proximal neck of the aneurysm [9]. However, whether the hypoxia is simply a terminal phenomenon seen in enlarged AAAs or is a key factor in the development of the aortic dilatation is debatable. Thus, in the present study we aimed to determine whether hypoperfusion of the VV could develop AAA.

To test this, we induced chronic hypoperfusion of the VV in the infrarenal abdominal aorta of rats and examined the resulting aneurysm development. We performed histological studies of the adventitial VV and used matrix-assisted laser desorption/ionization imaging mass spectrometry (MALDI-IMS) to assess tissue ischemia and hypoxia in the aortic walls. Furthermore, we studied 37 patients undergoing open repair of infrarenal AAA, including seven patients with small AAAs (diameter 30–49 mm), to determine whether the VV stenosis and resultant ischemia and hypoxia occur in the early stages of AAAs as well as in large AAAs (diameter ≥ 50 mm) of later stages.

## Materials and Methods

### Creation of aortic tissue hypoxia

**Fig 1:**
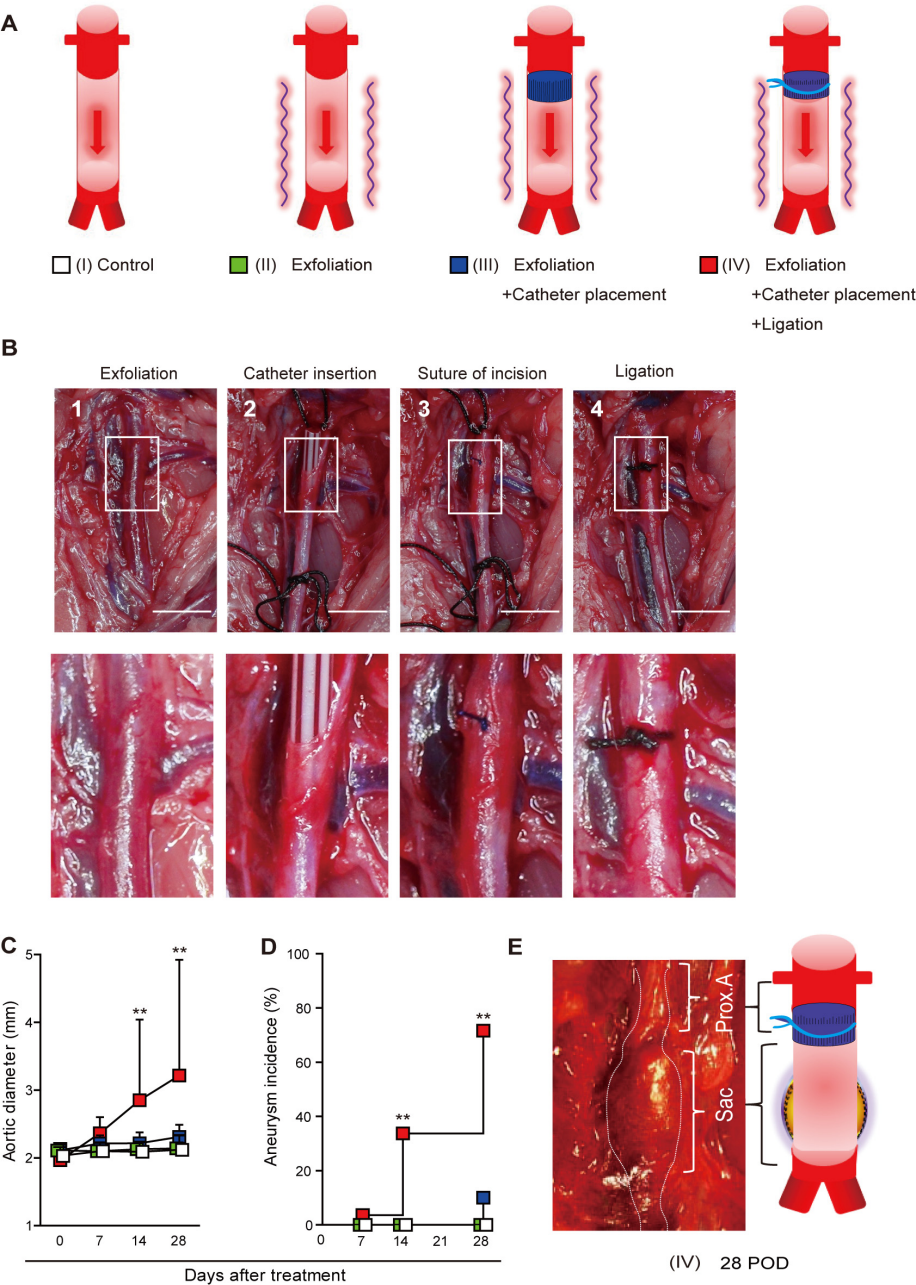
(A) Schematic of the four rat experimental groups. (n = 10 per each group). (B) Steps of the operation performed to induce abdominal aortic aneurysm (AAA) in a rat (group IV). The operation included the following steps: (1) the infra-renal aorta was exfoliated from the surrounding tissue; (2) a polyurethane catheter was inserted through a small incision in the aorta, and cut short to a 5 mm length; (3) the incision was repaired with a 10–0 monofilament suture and blood flow was restarted; (4) the aorta was ligated with a 4–0 silk suture over the inserted catheter. Scale bar = 5 mm. (C) Maximum aortic diameters measured with transabdominal ultrasonography. The aortic diameter steadily increased only in group IV rats. Aortic diameters are given as means ± standard deviation. **P < 0.01 by analysis of variance followed by Tukey’s post-test. (D) Incidence of AAA in rats. An aneurysm was defined as a more than 50% increase in the aortic diameter over baseline level. **P < 0.01 by Kaplan–Meier analysis. (E) Macroscopic view of a group IV rat’s aorta and the illustration on postoperative day 28, showing development of a fusiform AAA above the aortic bifurcation. Prox. A means proximal aorta between the renal artery and the ligation. Scale bar = 10mm.

**Fig 2:**
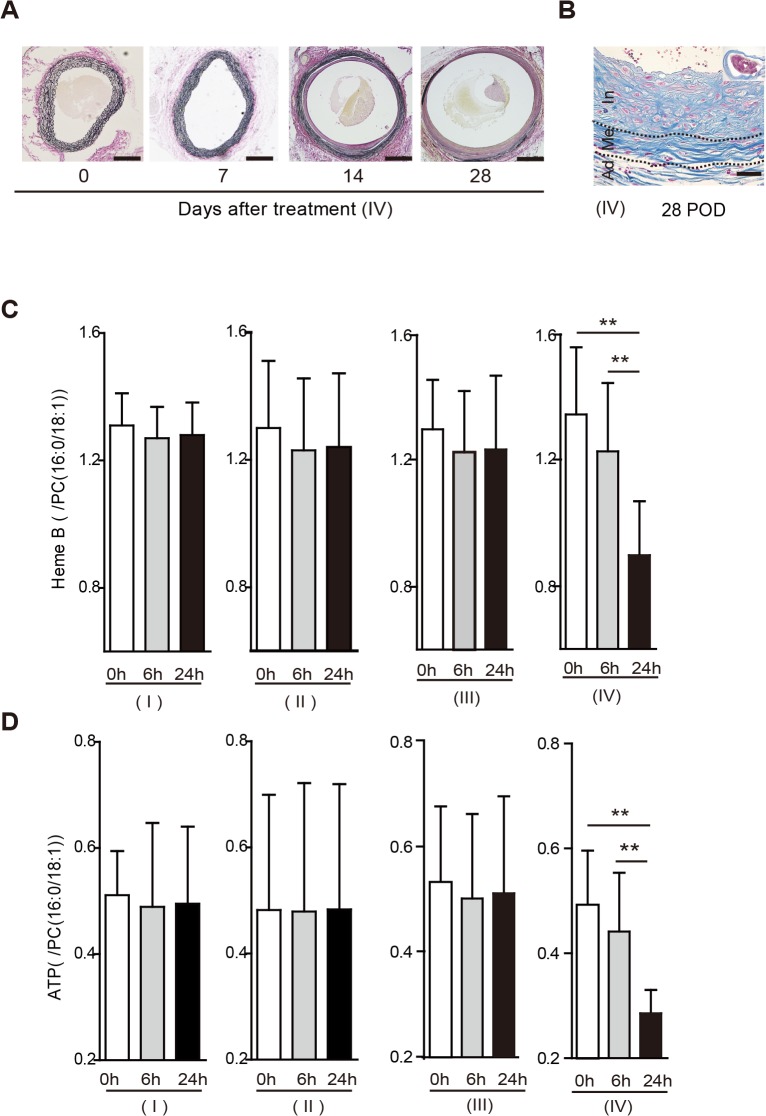
(A) Representative images of serial ultrasonographic studies (longitudinal and transverse views) of the abdominal aorta in group IV rats immediately after operation (0), and on postoperative days (POD) 7, 14, and 28. The aortic diameter gradually increased. An intraluminal thrombus was first seen at POD 14 and extended to the entire circumference at POD 28. Scale bar = 1mm. (B) Representative images of an aortic wall under high-power magnification with Elastica van Gieson staining in the abdominal aorta of group IV rats immediately after operation (0) and on postoperative days (POD) 7, 14, and 28. The medial elastic lamina became thinner over time. An intraluminal thrombus was first seen at POD 14 and had become thicker by POD 28. Scale bar = 100 μm.

**Fig 3:**
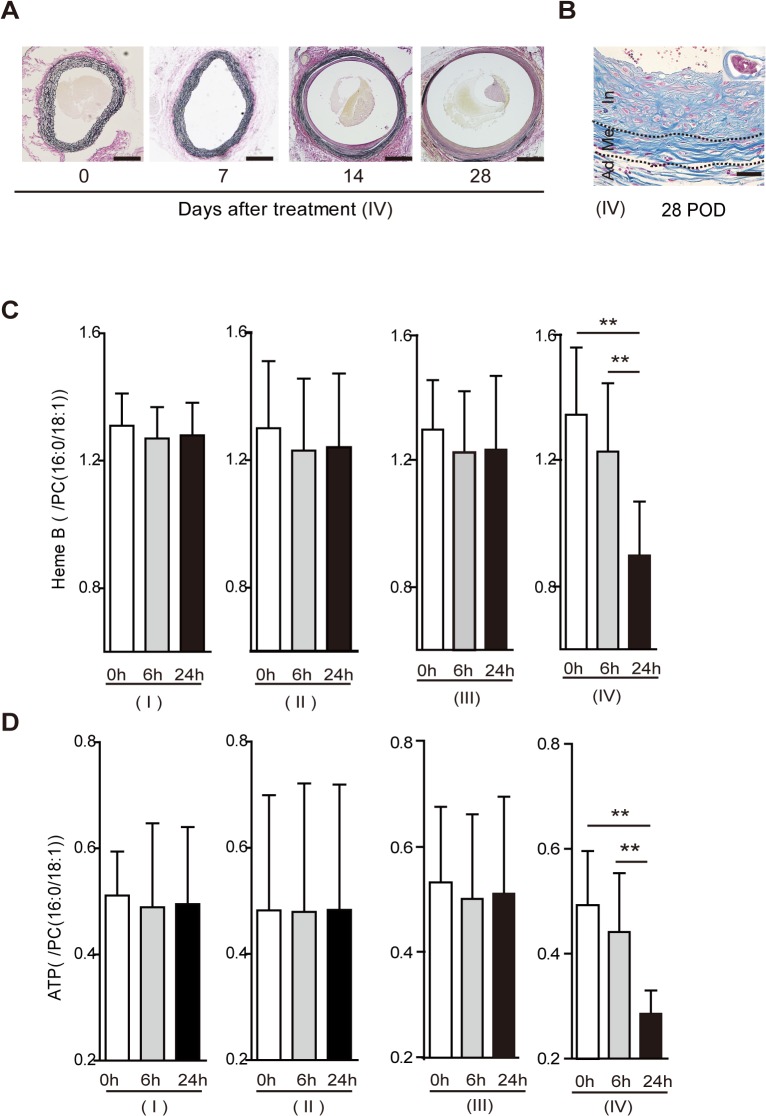
(A) Representative images of aneurysmal tissue with Elastica van Gieson staining in the abdominal aortae of group IV rats immediately after operation (0 days) and 7, 14, and 28 postoperative days (POD). As time passed, the aortic diameter gradually increased and the medial layer became thinner. Scale bar = 500 μm. (B) At POD 28, histological evaluation with Elastica van Gieson staining showed degeneration of the medial elastic lamina, sparse collagen fibers in the aortic adventitia, and marked intimal hyperplasia. Scale bar = 100μm. (C) Measurement of Heme B levels relative to those of phosphatidylcholine(PC16:0/18:1) with matrix-assisted laser desorption/ionization imaging mass spectrometry (MALDI-IMS) in the midpoint of the infra-renal aorta immediately (0h), 6 h, and 24 h after the operation in groups I—IV. MALDI-IMS showed that the ratio of Heme B to PC (16:0/18:1) in group IV had significantly decreased 24h after the operation. Results are the means ± standard deviation of three independent experiments. Statistical analysis was performed using analysis of variance for comparisons among the four groups. Post-hoc comparison was performed using Tukey’s test. **P<0.001 indicates a statistically significant difference. (D) Measurement of adenosine triphosphate (ATP) levels relative to those of PC (16:0/18:1) with MALDI-IMS at the midpoint of the infra-renal aorta immediately (0 h), 6 h, and 24 h after the operation in groups I—IV. MALDI-IMS showed that the ratio of ATP to PC (16:0/18:1) in group IV was significantly decreased at 24 h after the operation. Results are means ± standard deviation of five independent experiments. Statistical analysis was performed using analysis of variance for comparisons among the four groups. Post-hoc comparison was performed using Tukey’s test. **P<0.001 indicates a statistically significant difference.

**Fig 4:**
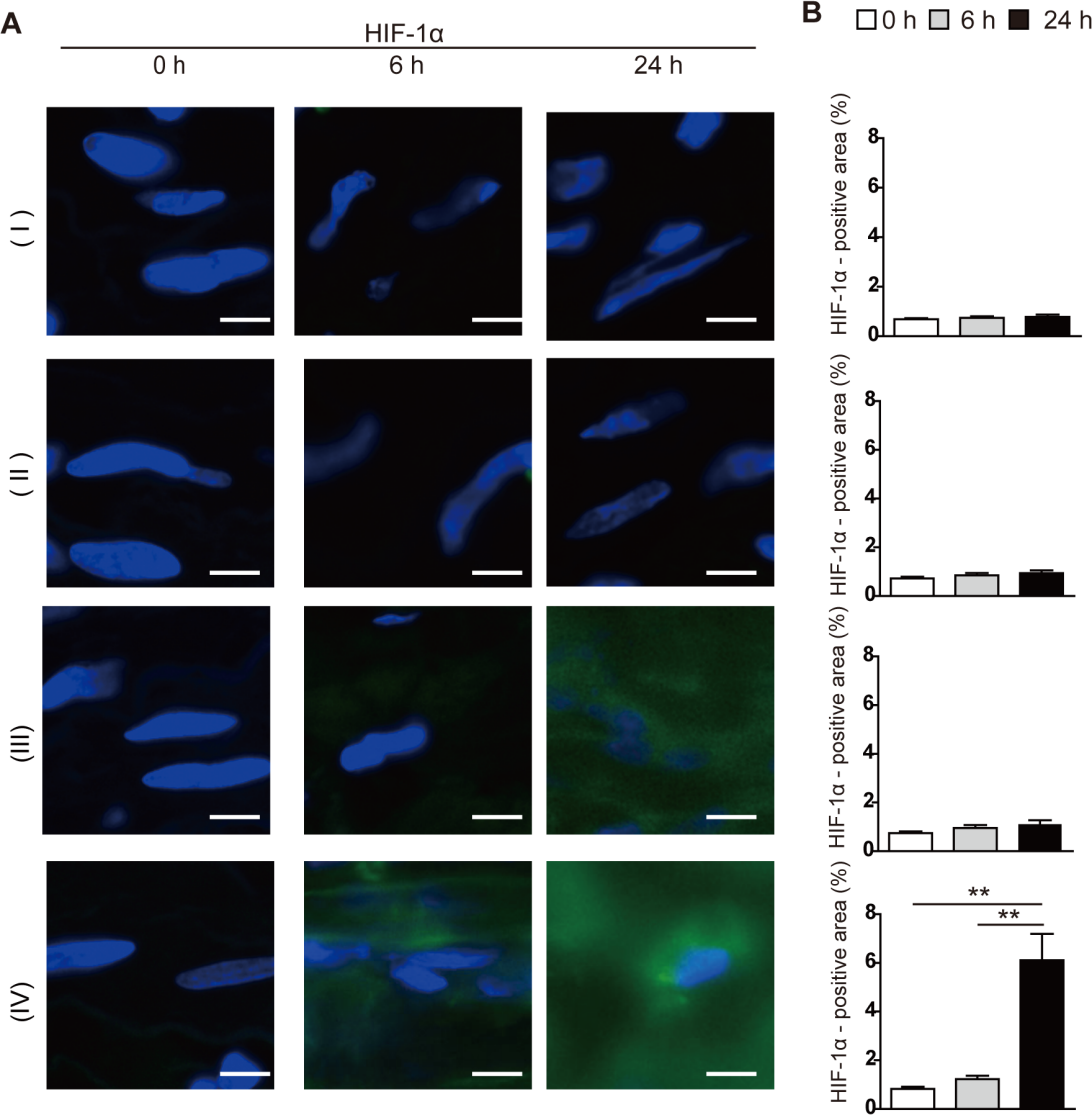
Immunohistochemical assay for hypoxia-inducible factor-1α (HIF-1α) in rat aortic tissue between the suture ligation and the aortic bifurcation in groups I—IV. (**A**) Nuclear and cytoplasmic expression of HIF-1α was observed in the media/adventitia at 24h after the operation in group IV. Scale bar = 100 μm. (**B**) HIF-1α-positive areas were counted per aortic section (n = 10, per group). Results are means ± standard deviation of five independent experiments at each time point. Statistical analysis was performed using analysis of variance for comparisons among the three groups. Post-hoc comparison was performed using Tukey’s test. **P<0.01 indicates a statistically significant difference.

To create localized aortic tissue hypoxia in rats, we tested four different groups (n = 10 each; Fig 1A). Rats in group I underwent laparotomy and were used as controls. In group II, the infra-renal aorta was exfoliated from the perivascular tissue (Fig 1B–1). In group III, the infra-renal aorta was exfoliated from the perivascular tissue as in group II; subsequently, a polyurethane catheter (23-gauge indwelling needle; Medikit: Supercath, Tokyo, Japan) was inserted via a small incision adjacent to the renal artery branches (Fig 1B–2) and then cut short to 5 mm in length. The incision was repaired with a 10–0 monofilament string (Fig 1B–3). In group IV, after the same procedures as in group III, the abdominal aorta was ligated with a 4–0 silk string together with the polyurethane catheter (Fig 1B–4).

### Sample collection

#### Rats

Animal care and experiments were performed in accordance with the guidelines of the Hamamatsu University School of Medicine animal care committee at the Center for Animal Care. Male Sprague—Dawley rats, weighing 620–800 g, were purchased from SLC (Shizuoka, Japan) and were provided food and water *ad libitum*. After the initial operation, the rats were re-laparotomized at the desired period to harvest aortic tissue. Aortic samples were frozen in isopentane and stored at-80°C. To assess the adenosine phosphate content in the aortic wall, liquid nitrogen was poured directly into the abdominal cavity to flash-freeze the viable tissue, and the aortic tissue was harvested and stored at-80°C until use. This method enabled assessment of the distribution of adenosine phosphate in the aortic wall by MALDI-IMS.

#### Humans

All procedures used in this study were approved by the clinical research ethics committee of Hamamatsu University School of Medicine. The study protocol was reviewed and approved by the university’s Ethics Committee of Clinical Research. The ethic committee’s approval number is 20372012.

Written informed consent for use of the aortic tissue samples was obtained from each patient. We enrolled 37 patients who underwent elective open surgery for repair of infra-renal AAA or bilateral common iliac artery aneurysm at the Division of Vascular Surgery, Hamamatsu University School of Medicine, between April 2008 and March 2012 (Table 1). The longitudinal aortic tissue between the renal artery and the bifurcation was dissected during the surgery for histological analysis. Six normal aortae obtained from cadavers were used as controls, with written informed consent being obtained from the donors’ families for use of the sample. The middle- portion of the abdominal aorta between the renal artery and the bifurcation was resected and collected during routine autopsies in the Department of Pathology at Hamamatsu University Hospital.

**Table 1:** Demographic and clinical data for patients with abdominal aortic aneurysm.

	Small AAA (30–49 mm)	Large AAA (≧ 50mm)
Sex (n) (male/female)	6 / 1	26 / 4
Age (years)	68.5 ± 5.2	70.2 ± 9.0
Height (m)	1.6 ± 0.1	1.6 ± 0.1
Weight (kg)	58.2 ± 6.1	58.0 ± 10.8
BMI (kg/m^2^)	22.5 ± 1.6	21.9 ± 2.4
Aortic diameter (mm)		
Infra-renal neck	20.5 ± 2.1	21.3 ± 2.2
Aneurysmal sac	37.8 ± 6.3	54.6 ± 12.2
Concomitant CIAA (n)	7	21
Maximum diameter (mm)	43.8 ± 8.0	28.1 ± 10.0
Serum TC (mg/dL)	186.3 ± 37.4	192.3 ± 30.8
HbA1c (%)	5.9 ± 0.8	5.5 ± 0.7
Ever smoked (n)	6	30

Values are mean ± standard deviation unless stated otherwise. Normal ranges: TC, 130–240 mg/dL; HbA1c, 4.3–5.8%. AAA, abdominal aortic aneurysm; CIAA, common iliac artery aneurysm; BMI, body mass index; TC, total cholesterol; HbA1c, glycosylated hemoglobin.

### Histological analysis

#### VV patency

Elastica van Gieson staining was used to stain the medial elastin. The luminal border and external elastic lamina (EEL) in each adventitial VV was traced at the same magnification. The size of the VV was measured as the total area bound by the EEL. Lumen patency of the VV was measured as the ratio of the luminal area to the total area bound by the EEL in each VV.

#### Smooth muscle cell depletion and elastin fragmentation

Medial elastin fragmentation and smooth muscle cell (SMC) depletion were scored as mild (1) to severe (5) using a histological grading system [10, 11]. Areas containing collagen fibers were quantitated for each aortic section.

### MALDI-IMS

Samples were prepared as previously described [12, 13], except for certain modifications. In brief, abdominal aortae were cut with a cryostat (CM1950; Leica, Wetzlar, Germany) into 8-μm-thick sections. The sections were then mounted on glass slides coated with indium-tin oxide (Bruker Daltonics, Bremen, Germany). Then, a 50 mg/mL solution of 2,5-dihydroxybenzoic acid in methanol/water (7:3, v/v) was sprayed onto the sections with an airbrush with a 0.2-mm nozzle caliber (Procon Boy FWA Platinum; GSI Creos, Tokyo, Japan). IMS was performed using a MALDI time-of-flight type instrument (Ultraflex II, Bruker Daltonics) equipped using a 355-nm Nd:YAG laser at a repetition rate of 200 Hz. Data were acquired with a step size of 20 μm for samples in the positive ion mode (reflector mode); The *m/z* ratios in the range of 400–1000 were measured for Heme B, and ratio of 50–400 was measured for adenosine triphosphate (ATP). The laser diameter was set to the minimum. All spectra were acquired automatically using FlexImaging software (Bruker Daltonics), which was also used to create two-dimensional ion-density maps.

### Identification of biomolecules

To identify the spectral peaks, tandem mass spectrometry was performed using a MALDI-quadrupole ion trap—time of flight mass spectrometer (AXIMA-QIT; Shimadzu, Kyoto, Japan) [14]. The specific fragment patterns of phosphatidylcholine (PC), cholesterol ester, Heme B and ATP were identified according to previous reports [9] [15].

### Serial aortic diameter determination using ultrasound imaging

After the operation, the maximum diameter of the abdominal aorta of each rat was measured using ultrasound (Veno770, VisualSonics, Toronto, Canada), which was performed 7,14, and 28 days after the operation.

### Immunohistochemistry

Abdominal aortae were harvested and cut with a cryostat (CM1950; Leica) into 8-μm-thick sections. The sections were fixed with 4% paraformaldehyde in phosphate-buffered saline (PBS; pH 7.4) for 10 min at room temperature. For conventional immunostaining, the slides were incubated at room temperature with primary antibodies: anti-SMC (1:50, Thermo Fisher Scientific, Waltham, MA, USA), anti-CD68 (1:100, Abcam, Cambridge, UK), anti-MMP-2 (1:100, Daiichi Fine Chemical, Toyama, Japan), anti-MMP-9 (1:100, Abnova, Taipei, Taiwan), and rabbit anti-HIF-1α) (1:100; Novus Biologicals, Littleton, CO, USA) for 120 min. The immune complexes were then detected with a polymer reagent (Histofine Simple Stain MAX PO; Nichirei Bioscience, Tokyo, Japan). The binding of primary antibodies to their specific antigens was visualized using the diaminobenzidine kit (Dako, Carpinteria, CA, USA) and imaged on a light microscope equipped with a Nikon Eclipse 80i camera using Lumina Vision software (ver. 3.0, Mitani, Tokyo, Japan). Macrophages within the aortic wall were counted as CD68^+^ cells per aortic cross-section.

### Immunofluorescence staining

Tissue sections (8 μm) were fixed with 4% paraformaldehyde in PBS (pH 7.4) for 10 min at room temperature and stained routinely with Elastica van Gieson staining for elastin, and picrosirius red (PSR) for collagen. After rinsing with PBS, the sections were preincubated with 10% normal goat serum (Nichirei Biosciences, Tokyo, Japan) and incubated overnight at 4°C with mouse anti-CD68 and rabbit anti-MMP-9. Immunoreactivity was visualized using Alexa Fluor 488-conjugated anti-rabbit immunoglobulin G (Molecular Probes, Invitrogen, Carlsbad, CA, USA) and Alexa Fluor 594-conjugated anti-mouse immunoglobulin G. All Alexa Fluor-conjugated secondary antibodies were diluted 200-fold. The slides were mounted in a glycerol-based Vectashield medium (Vector Laboratories, Burlingame, CA, USA) containing the nucleus-staining reagent 4’,6-diamidino-2-phenylindole.

### Histological morphometry

Digital images were obtained using an Olympus BX51 light microscope. We classified the sections into four categories according to the severity of both elastic disruption and SMC depletion as described below[16]. Normal media of the abdominal aorta have intact elastic lamina without any signs of elastic strand breaks or degradation. A grade of 1 indicates a mild disruption of the medial-layer elastic network, with only the EEL disrupted. A grade of 2 signifies moderate disruption of the medial-layer elastic network, with both the EEL and outer media-layer broken or disrupted. A grade of 3 denotes high disruption of the medial elastic network, with the EEL and both medial elastic layers showing breakage and/or degradation. A grade of 4 indicates severe disruption of the medial-layer elastic network, with all four elastic layers showing signs of breakage and/or degradation.

Next, the number of CD68^+^ cells was determined by counting all immunostained cells per aortic cross section.

Collagen content was evaluated using picrosirius red staining, and levels of HIF-1α, MMP-2, and MMP-9 were evaluated using immunohistochemical staining[16, 17]. Images of sections stained for collagen, MMP-2, and MMP-9 were analyzed using Image J software (National Institutes of Health, Bethesda, MD, USA). We evaluated three cross-sections from each tissue sample, taken at 100-μm intervals, and averaged the results. We set a threshold by which to compute the area positive for each protein and then calculated the ratio of the positive area to the total aortic area.

### *In situ* gelatin zymography

Low-gelling-temperature agarose (1%; Sigma-Aldrich, St. Louis, MO, USA) was dissolved in PBS at 80°C and then cooled to 37°C. Dye-quenched gelatin (DQG; Invitrogen, Carlsbad, CA) was diluted to 1 mg/mL in distilled water; this solution was then further diluted 1:10 in the low-gelling-temperature agarose solution. The agarose solution containing DQG (40μL) was then applied to the top of one of two tissue sections, which was then covered with a microscope slide coverslip; agarose solution without DQG (40μL) was applied to the second tissue section so that tissue autofluorescence could be measured. The sections were then stored at 4°C for 30 min to allow the agarose to gel; after this, they were kept at room temperature for 4 h to allow the tissue gelatinases to incubate with the DQG substrate. The sections were then imaged using a fluorescence microscope at an excitation wavelength of 480 nm and 20× magnification. Autofluorescence sections were imaged at an exposure time of 500 ms; DQG-containing sections were imaged at an exposure time of 50 ms. The intensity of each area was quantified using Scion Image software (Scion, Austin, TX, USA). We set a threshold by which to compute the area positive for each enzyme and then calculated the ratio of the positive area to the total area of each section.

### Liquid chromatography-tandem mass spectrometry

Frozen rat aortae were homogenized in methanol (50 mL per 10 mg tissue) using a bead homogenizer (Micro Smash MS-100R; Tomy, Tokyo, Japan), followed by the addition of an equal volume of chloroform and 0.4 times the volume of Milli-Q water. After centrifugation (3 cycles at 4,000 rpm for 60 s), the aqueous phases were filtered using an ultrafiltration tube (Ultrafree- MC, Millipore, Billerica, MA, USA), and the filtrate was dried. The dried residues were redissolved in 50 μL Milli-Q water and were used for mass spectrometry analysis. To measure the concentration of hydrophilic nucleotides (ATP, adenosine diphosphate [ADP], adenosine monophosphate [AMP], adenine dinucleotide phosphate [NADP], and reduced NADP [NADPH]), liquid chromatography–tandem mass spectrometry was performed by a previously reported method[18]. Briefly, a triple-quadrupole mass spectrometer equipped with an electrospray ionization (ESI) ion source (4000 QTRAP, AB Sciex, Foster City, CA, USA) was used in the negative-electrospray ionization and multiple reaction monitoring modes. The samples were resolved using an Acquity UPLCBEH C18 chromatography column (Waters, Milford, MA, USA; 100 mm × 2.1 mm internal diameter, particle size 1.7μm) and separated by use of two mobile phases A (10 mM tributylamine aqueous solution with pH adjusted to 4.95 using 15mM acetic acid) and B (methanol), at a flow rate of 0.2 mL/min. Ion transitions from *m/z* 506 to *m/z* 79, *m/z* 426 to *m/z* 79, and *m/z* 346 to *m/z* 79 were monitored for quantification of ATP, ADP, and AMP, respectively. For NADP and NADPH, ion transitions from *m/z* 742 to *m/z* 620 and from *m/z* 744 to *m/z* 79, respectively, were monitored.

### Statistical analysis

The results of the experiments were analyzed using StatView software (version 5.0; SAS Institute, Cary, NC, USA). All data are expressed as means values ± standard deviation. Statistical analysis was performed using analysis of variance for comparisons of VV patency among human aorta samples and changes in aortic diameter among rats. Post-hoc comparisons were performed using Tukey’s test. Comparisons between the proximal aorta and the AAA sac were performed using the paired *t*-test. Incidence of aneurysm in the rat model was analyzed using Kaplan–Meier analysis.

## Results

### Aneurysm development resulting from induced localized abdominal aortic VV hypoperfusion in rats

Four experimental groups of rats were tested, as follows (Fig 1A). Group I was the control rats, Group II consisted of rats that underwent exfoliation of the infrarenal abdominal aorta from the surrounding tissue. Group III consisted of rats undergoing exfoliation plus catheter insertion into the infrarenal abdominal aorta. Group IV consisted of rats undergoing exfoliation, catheter insertion into the infrarenal abdominal aorta, and aortic ligation (Fig 1B). All rats survived throughout the experiments. Among the groups, only group IV showed a steady increase in aortic diameter up to postoperative days (POD) 28 (Fig 1C), with a nearly 80% incidence of AAA (Fig 1D). The other groups developed no aneurysm. Serial ultrasounds showed that an ILT first appeared on POD 14 and extended to the entire circumference of the aorta by POD 28 (Fig 2A); these observations were confirmed histologically (Fig 2B). The AAA developed in a fusiform shape between the aortic ligation and the aortic bifurcation (Fig 1E). Histological examination demonstrated that there was no change in the aortic wall below the ligation immediately after the operation in group IV; however, the medial layer became thinner as time passed (Figs 2B and 3A). In addition, elastic fiber fragmentation in the aortic media and sparse collagen fibers in the aortic adventitia were observed at POD 28 in group IV (Fig 3B). Immunohistochemical examination of group IV rats showed nuclear and cytoplasmic expression of HIF-1α in the media and adventitia 24h after operation (Fig 4A and 4B).

**Fig 5:**
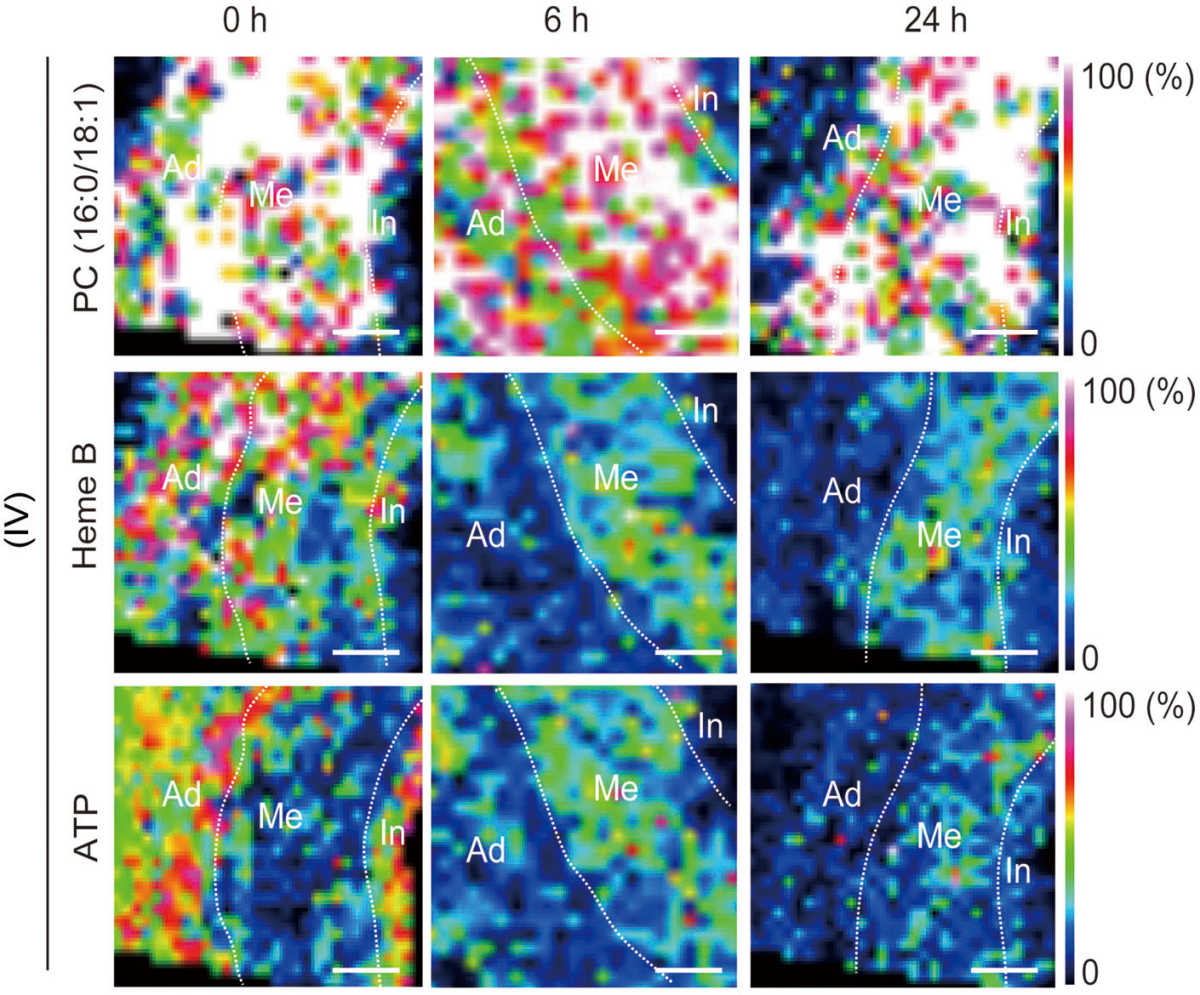
Representative images obtained by matrix-assisted laser desorption /ionization imaging mass spectrometry (MALDI-IMS) of the abdominal aorta between the infra-renal ligation and the aortic bifurcation in group IV rats. Distributions of phosphatidylcholine (PC;16:0/18:1), Heme B, and adenosine triphosphate (ATP) in the aortic tissue were visualized. PC (16:0/18:1) was observed ubiquitously immediately after (0) the operation and at 6 h and 24 h following the operation. On the other hand, both Heme B and ATP in the outer media and the adventitia decreased at 6 h and 24 h after the operation. Ad, adventitia; Me, media; In, intima. Scale bar = 100 μm.

To evaluate the tissue blood perfusion, we assessed Heme B in the aortic wall. Heme B is a specific marker for blood and is used to indicate tissue blood flow status[14, 19]. Decreased levels of ATP and Heme B in the outer medial and adventitial regions were identified in group IV by using MALDI-IMS, which can be used to profile discrete cellular regions and obtain region-specific images, providing information on the relative abundance and spatial distribution of proteins, peptides, lipids, and drugs (Fig 5) [20]. The ion intensities of Heme B and ATP on MALDI-IMS relative to that of phosphatidylcholine (PC) (16:0/18:1), which was ubiquitous throughout the aortic wall at all postoperative time points, were used for the comparison of the tissue contents of both Heme B (Fig 3C) and ATP (Fig 3D). In group IV, both Heme B and ATP were significantly lower 24h after the operation than immediately afterwards.

### Biological features of AAAs resulting from localized abdominal aortic VV hypoperfusion in rats

**Fig 6:**
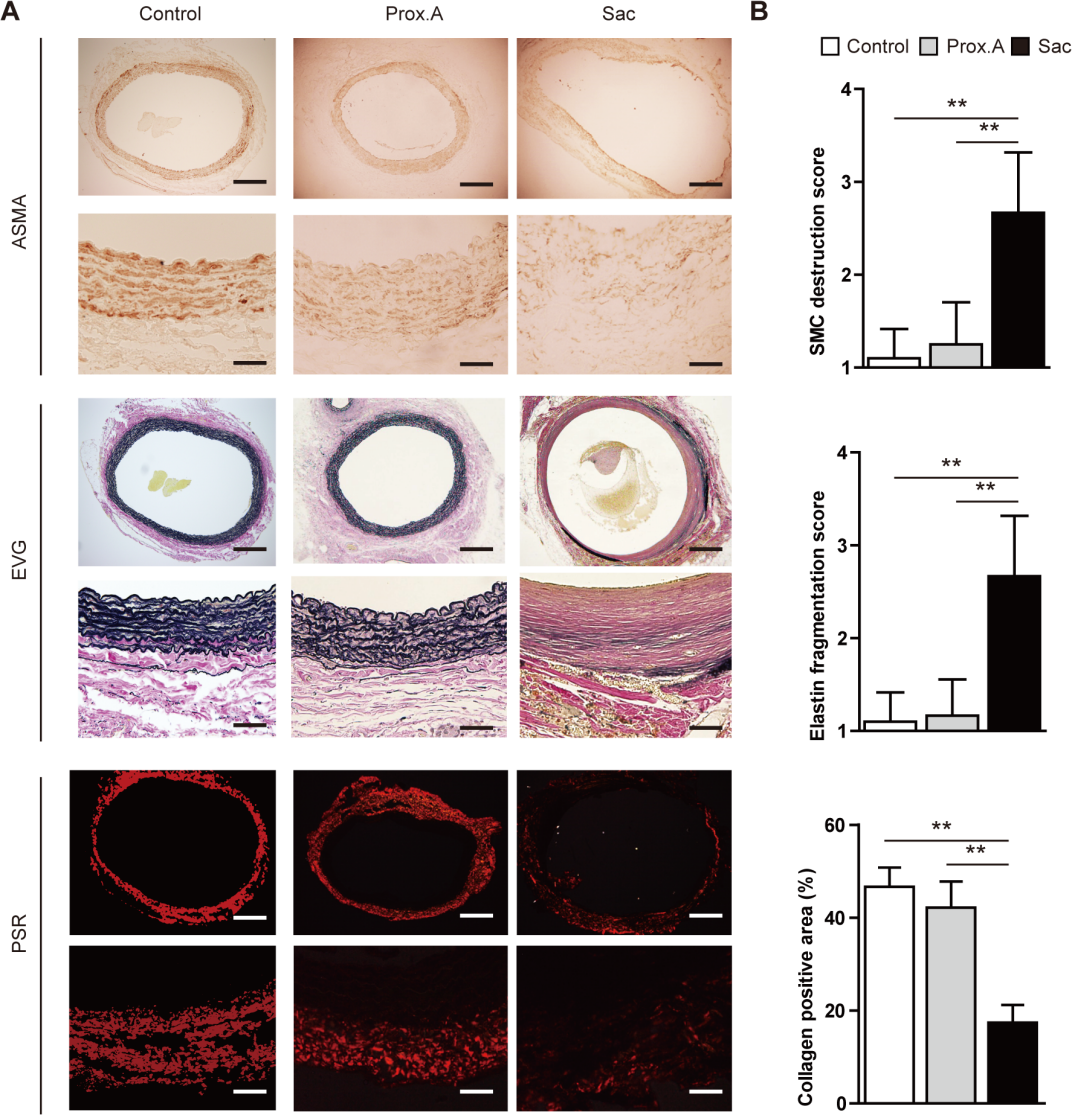
Characterization of rats that underwent polyurethane catheter insertion and aortic ligation resulting in aortic aneurysms (group IV). Data were collected using aortic specimens harvested on postoperative day 28 (n = 10). Group I was used as a control group. The specimens included infra-renal proximal aorta (Prox.A) and aneurysmal sac (Sac). All data are expressed as means ± standard deviation. (**A**) Representative photomicrographs of smooth muscle cells (SMCs; alpha-smooth muscle cell actin, ASMA), elastin (Elastica van Gieson staining, EVG), and collagen fibers (picrosirius red; PSR). Scale bar in upper panels = 500 μm, in lower panels = 100 μm. (**B**) Quantitative analysis of SMC, elastin fragmentation and collagen distribution. SMC depletion was scored as mild (1) to severe (5) using a histological grading system. Areas positive for collagen fibers were quantitated per aortic section. Results are means ± standard deviation of three independent experiments. Statistical analysis was performed using analysis of variance for comparisons among the three groups. Post-hoc comparison was performed using Tukey’s test. **P<0.01 indicates a statistically significant difference.

**Fig 7:**
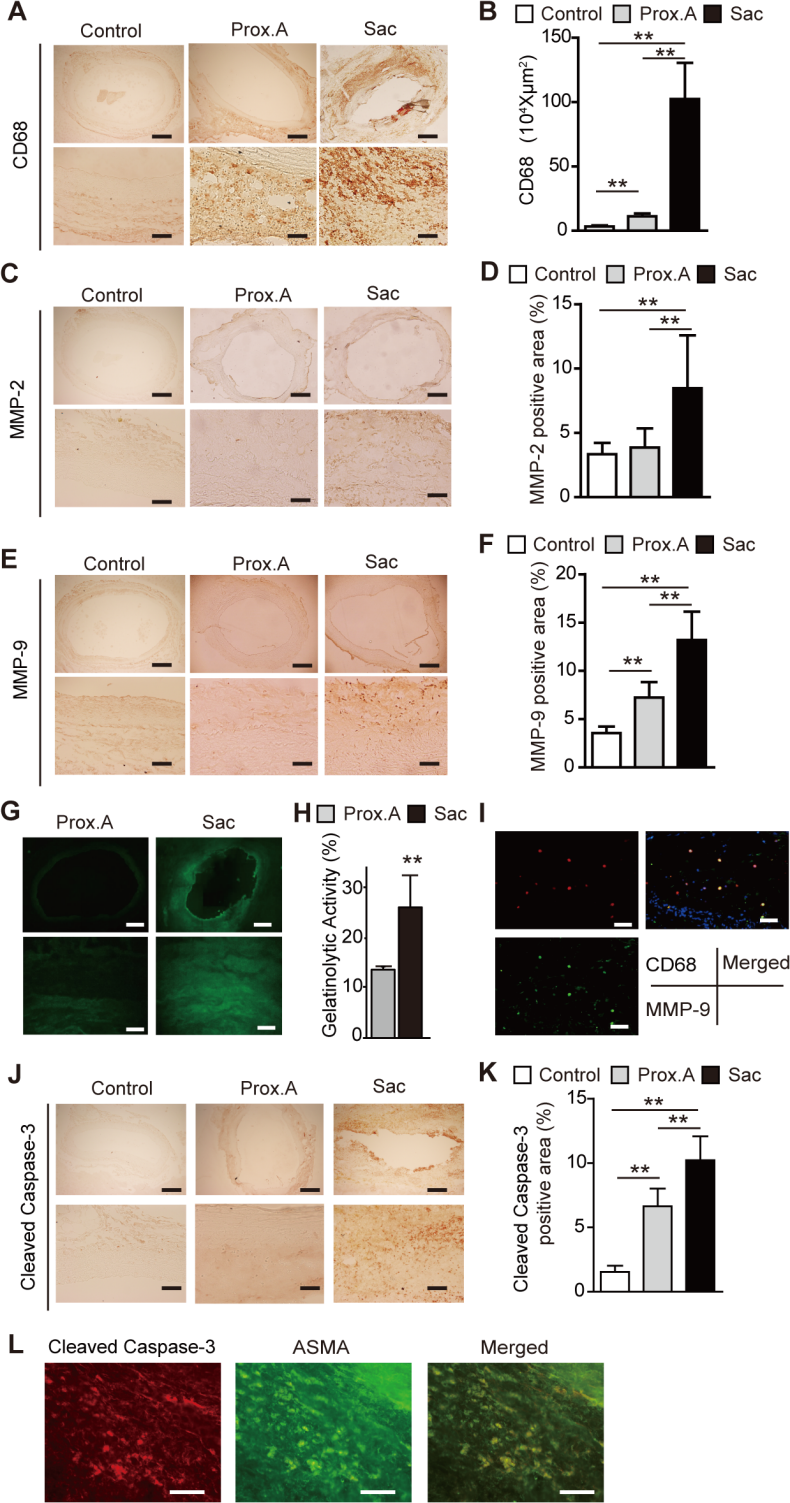
(A) Representative photomicrograph of macrophage (CD68+) infiltration. Scale bar in upper panels = 500 μm, in lower panels = 100 μm. (B) Macrophages within the aortic wall were counted as CD68+ cells. (C) (E) Aortic specimens were stained with antibodies against matrix metalloproteinase MMP-2 and MMP-9. Scale bar in upper panels = 500 μm, in lower panels = 100 μm. (D)(F) Areas positive for MMP-2 and MMP-9 were counted per aortic section (n = 10 per group). Statistical analysis was performed using analysis of variance for comparisons among the three groups. Post-hoc comparison was performed using Tukey’s test. **P<0.01 indicates a statistically significant difference. (G)(H) Representative examples and semiquantitative analysis (percent positive relative to the total area of the section) of gelatinase activity (green) in aortic aneurysms (group IV). Scale bar in upper panels = 500 μm, in lower panels = 100 μm. The experiment was repeated three times. **P < 0.01 by paired *t*-test. (I) Representative images of immunofluorescence staining for MMP-9 (green), CD68+ cells (macrophages;red), and 4ʹ,6-diamidino-2-phenylindole (blue). Merged images showed that MMP-9 was secreted from macrophages. Scale bar = 20 μm. (J) Representative photomicrograph of cleaved caspase-3. Scale bar in upper panels = 500 μm, in lower panels = 100 μm. (K) Cleaved caspase-3+ cells within the aortic wall were counted. Statistical analysis was performed using analysis of variance for comparisons among the three groups. Post-hoc comparison was performed using Tukey’s test. **P<0.01 indicates a statistically significant difference.

Histologically, the proximal aortic wall and sac in group IV were compared to that in group I (control group) on POD 28, which revealed marked degeneration and disruption of the elastic lamina with significantly fewer SMCs in the sac than in the proximal abdominal aorta just below the renal arteries and control group (Fig 6A and 6B). Macrophage infiltration was higher in the sac than in normal tissue from the adventitia to the media (Fig 7A and 7B). The levels of MMP-2 and MMP-9 in the sac, which are elevated in human AAAs, were likewise significantly higher than in the proximal abdominal aorta in both immunohistochemistry (Fig 7C–7F) and *in situ* zymography for gelatinase activity (Fig 7G and 7H). Mearged images of the immunofluorescence staining showed that MMP-9 had been secreted from CD68^+^ cells, identifying the infiltrating macrophages as the source of the MMPs (Fig 7I).

### Adventitial VV stenosis and perturbation of tissue oxygen metabolism resulting from induced localized abdominal aortic VV hypoperfusion in rats

**Fig 8:**
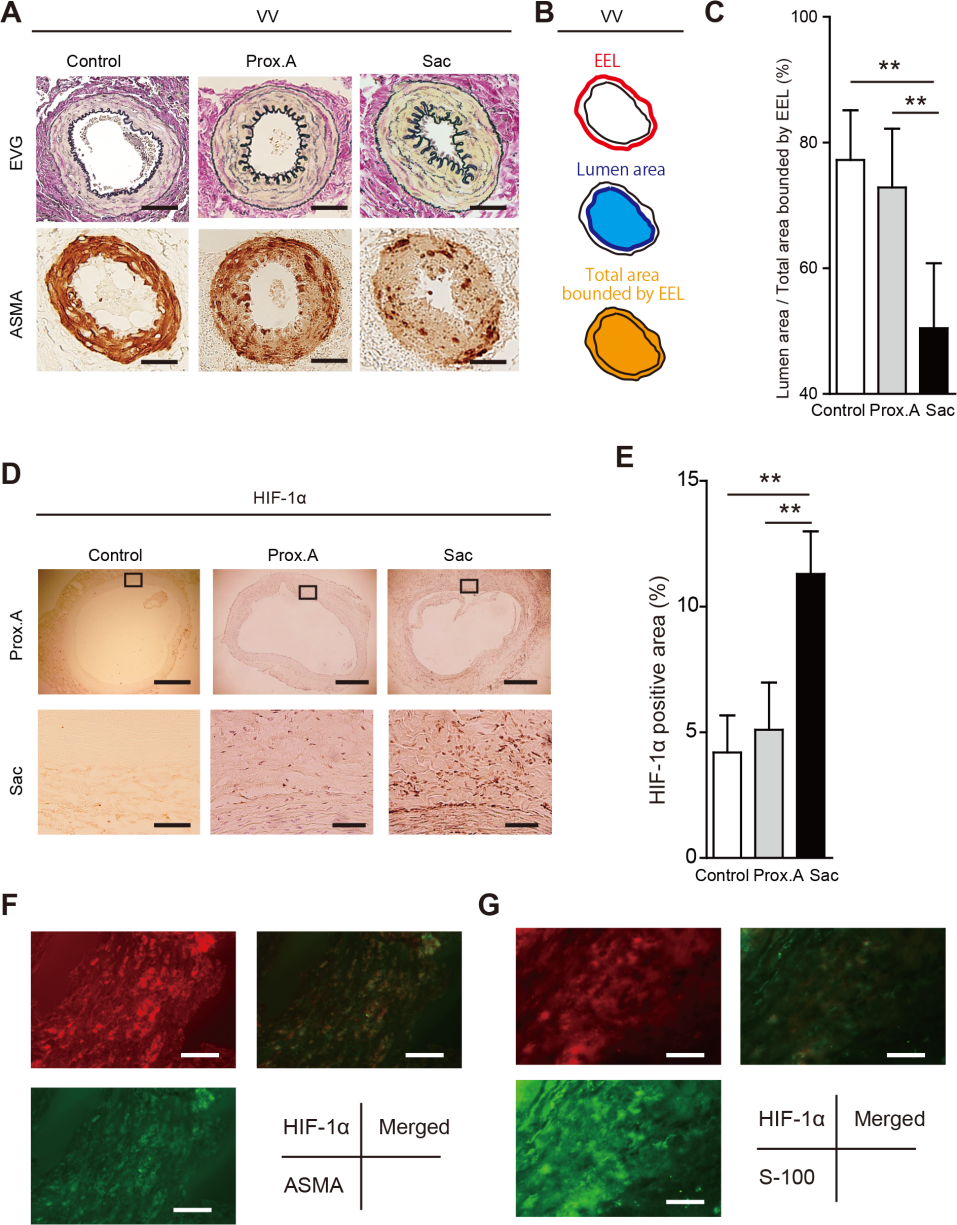
Tissue ischemia and hypoxia in aortic aneurysms induced by a polyurethane catheter insertion and aorta ligation in rats (group IV). Data were collected using aortic specimens recovered at postoperative day 28 (n = 10). The specimens included group I (Control), infra-renal proximal aorta specimens (Prox.A) and aneurysmal sac specimens (Sac). All data are expressed as means ± standard deviation. (**A**) Representative adventitial vasa vasorum (VV) with Elastica van Gieson (EVG) staining and alpha-smooth muscle cell actin (ASMA) staining in Prox.A and Sac. Scale bar = 10 μm. (**B**) Schematic of the measurement method for lumen patency of VV. (**C**) Lumen patency of VV was measured as the ratio of lumen area to the total area of the VV which was bound by the external elastic lamina (EEL) in each VV. Comparison of the lumen patency of VV between Prox. A and Sac showed significant stenosis in Sac VV. Results are means ± standard deviation of six independent experiments. (**D**) Immunohistochemistry for HIF-1α in Control, Prox. A, and Sac. The squared area in the upper panels is magnified in lower panels. Scale bar in upper panels = 500 μm, in lower panels = 100 μm. (**E**) HIF-1α-positive areas were counted per aortic section (n = 10, each group). Results are means ± standard deviation of six independent experiments. Statistical analysis was performed using analysis of variance for comparisons among the three groups. Post-hoc comparison was performed using Tukey’s test. **P<0.01 indicates a statistically significant difference. (**F**) Representative images of immunofluorescence staining for smooth muscle cells (SMCs; alpha-smooth muscle cell actin, ASMA; green) and HIF-1α (red). Merged images showed that HIF-1α was positive in the medial SMCs. Scale bar = 20 μm. (**H**) Representative images of immunofluorescence staining for fibroblasts (S-100) (green) and HIF-1α (red). Merged images showed that HIF-1α was positive in the adventitial fibroblasts. Scale bar = 20 μm.

**Fig 9:**
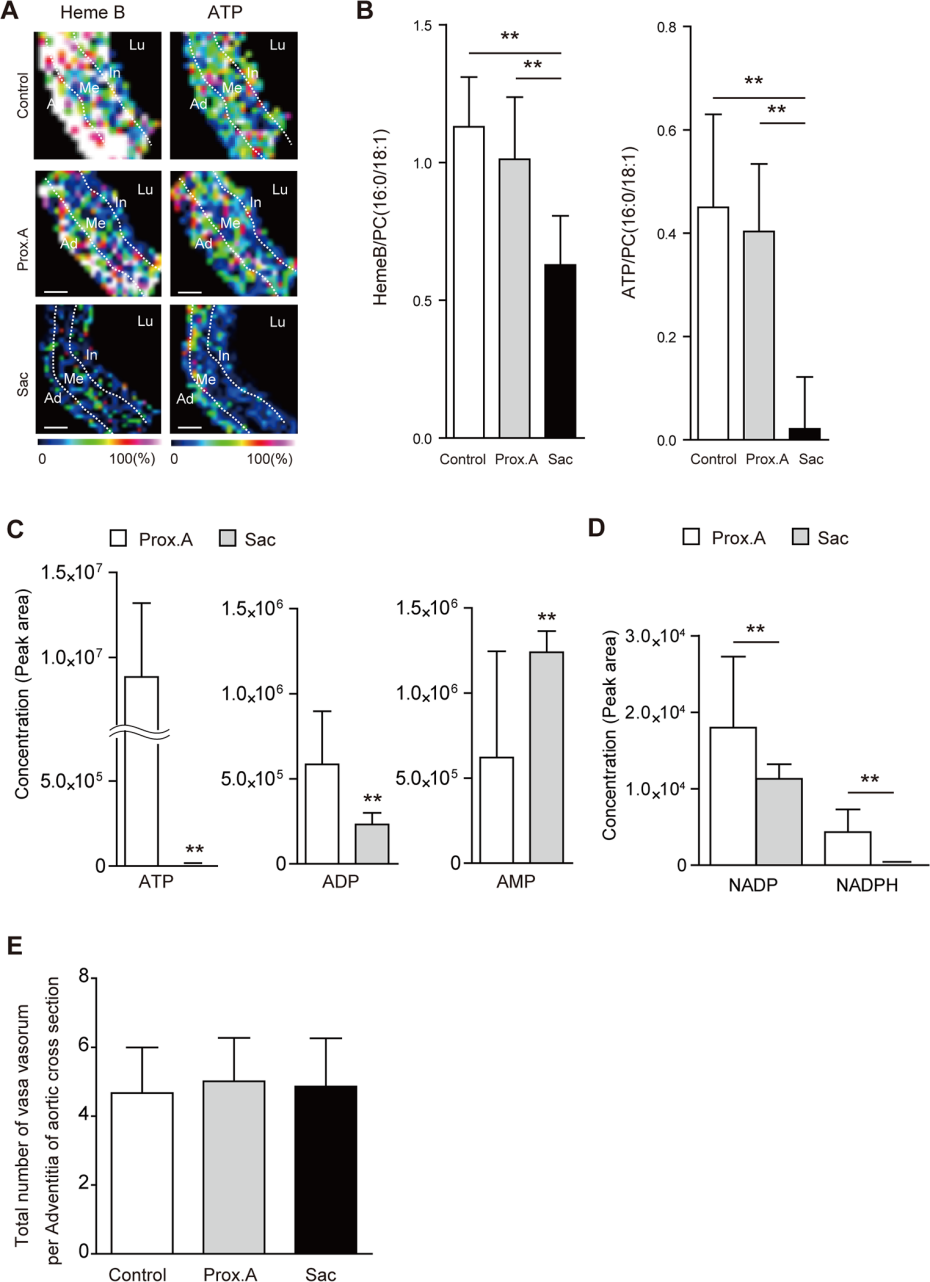
Tissue oxygen metabolism in rats (Group IV). (**A**) Representative matrix-assisted laser desorption/ionization imaging mass spectrometry (MALDI-IMS) image showing the distribution of Heme B and adenosine triphosphate (ATP) in Prox.A and Sac. (**B**) Comparison of the intensity of HemeB relative to PC (16:0/18:1) between Prox.A and Sac. **P < 0.01. Results are means ± standard deviation of three independent experiments. (**C**) Comparison of tissue levels of ATP, adenosine diphosphate (ADP), and adenosine monophosphate (AMP) between Prox.A and aneurysmal sac specimens (Sac). **P < 0.01 by paired *t*-test. Results are means ± standard deviation of five independent experiments. (**D**) Comparison of tissue levels of nicotin-amide adenine dinucleotide phosphate (NADP) and reduced NADP NADPH between Prox.A and Sac. **P < 0.01 by paired *t*-test. Results are means ± standard deviation of five independent experiments. (**E**) Comparison of the the number of vasa vasorum per adventitia of the aortic cross sections among the three groups. Results are means ± standard deviation of three independent experiments. Statistical analysis was performed using analysis of variance for comparisons among the three groups. Post-hoc comparison was performed using Tukey’s test.

By POD 28, the VV in the aneurysmal sac in group IV showed stenosis with proliferation of SMCs (Fig 8A). Dividing the luminal area of the VV by their total area, which was bound by the EEL in each VV, was calculated for the comparison. The results showed that the adventitial VV in the aneurysmal sac was significantly stenotic in comparison with the proximal abdominal aorta (Fig 8B and 8C). Nuclear expression of HIF-1α was observed in the media and adventitia of the aneurysmal sac, whereas no HIF-1α expression was observed in the proximal aorta (Fig 8D and 8E). HIF-1α was positively expressed in the medial SMCs, which was confirmed by the double immunostaining with the SMC marker alpha-smooth muscle cell actin (ASMA; Fig 8F). HIF-1α was also positively expressed in the adventitial fibroblasts in the sac, which was confirmed with the double immunostaining with the fibroblast marker S-100 (Fig 8G). MALDI-IMS revealed that tissue distributions of both Heme B and ATP were decreased in the outer media and adventitia of the sac relative to the proximal abdominal aorta (Fig 9A **)**, and the levels of tissue contents were significantly decreased (Fig 9B). Moreover, liquid chromatography-tandem mass spectrometry identified that the levels of ATP, ADP, NADP, and NADPH were all lower in the sac than in the proximal abdominal aorta, whereas levels of AMP were higher (Fig 9C and 9D). In addition, the numbers of adventitial VVs per whole area of the aortic cross section among the three groups was compared. The results indicated that the number of VVs was not significantly different among the three groups (Fig 9E).

### Occurrence of VV stenosis and hypoperfusion in human AAA wall

**Fig 10:**
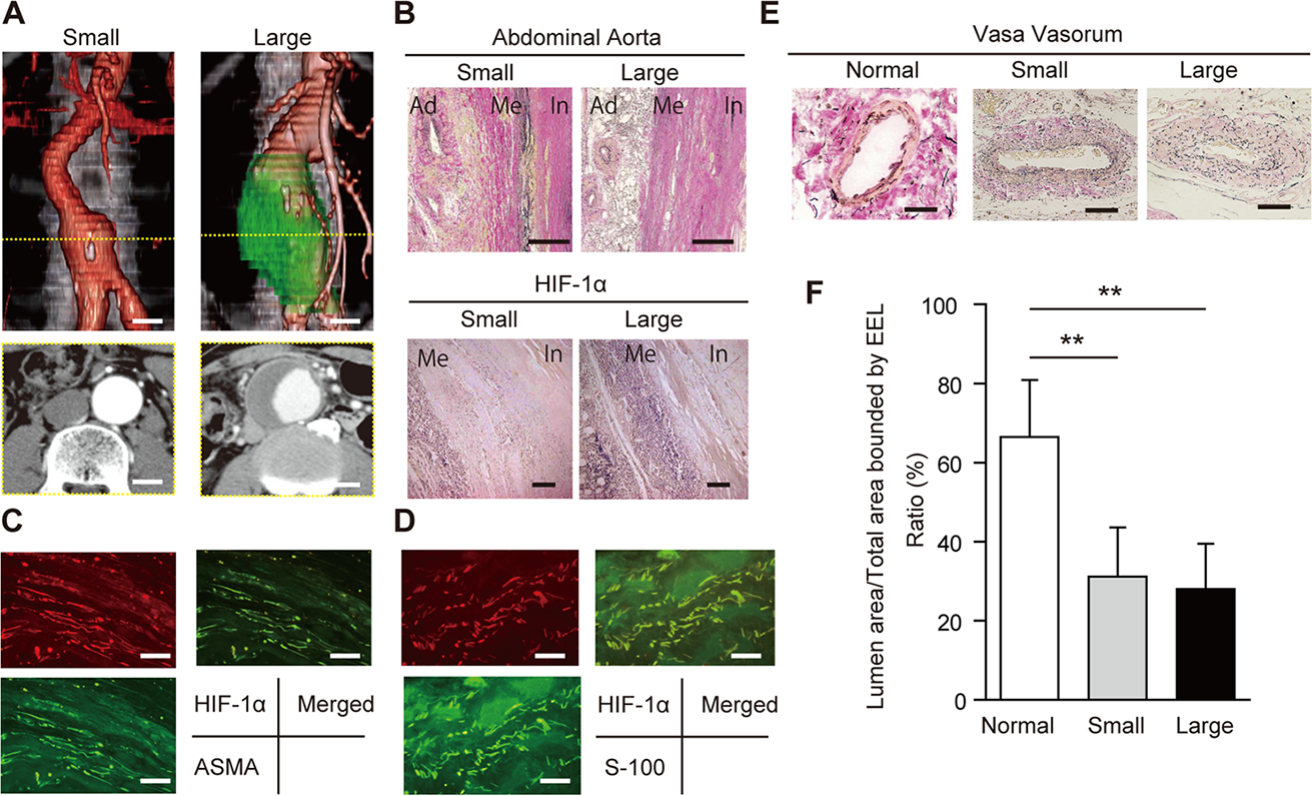
(A) Preoperative contrast-enhanced 3D-multiple-detector computed tomographic images of a patient with an abdominal aortic aneurysm (AAA). Scale bar = 20 mm. AAAs 30–49 mm in diameter were classified as small, and those more than 50 mm in diameter were classified as large. (B) Upper: representative images of cross-sections of small and large AAAs, (Elastica van Gieson staining). In the small AAAs, medial elastic fibers are relatively well preserved in comparison with the large AAAs. In these large AAAs, the medial elastic lamina is extensively disrupted, with a thick intraluminal thrombus. Ad;,adventitia; Me, media; In, intima. Scale bar = 500 μm. Lower part: representative images from the immunohistochemical assay for hypoxia-inducible factor-1α (HIF-1α) in a small and a large AAA. Nuclear and cytoplasmic expression of HIF-1α was observed in the media/adventitia in both AAAs. Scale bar = 500 μm. (C) Representative images of immunofluorescence staining for smooth muscle cells (SMCs; alpha-smooth muscle cell actin, ASMA) (green) and HIF-1α (red). Merged images showed that HIF-1α was positive in SMCs. Scale bar = 20 μm. (D) Representative images of immunofluorescence staining for fibroblasts (S-100; green) and HIF-1α (red). Merged images showed that HIF-1α was positive in fibroblasts. Scale bar = 20 μm. (E) Patency of the adventitial vasa vasorum (VV) in human tissues (Elastica van Gieson staining). The VVs are patent in the normal aorta but stenotic in the small and larger AAA sacs. Scale bar = 50μm. (F) Lumen patency of the VV was measured as the ratio of the lumen area to the total area, which was bounded by the external elastic lamina (EEL) in each VV. The figure compares lumen patency of adventitial VV among a normal aorta, a small AAA sac, and a large AAA sac adventitial VV. Data were obtained from six cadavers with normal aortae, seven patients with small AAAs, and 30 patients with large AAAs). **P < 0.01.

We next determined whether VV stenosis and the resultant hypoperfusion occur in the early stages of human AAAs (i.e., small AAA) as well as in more advanced large AAAs. Seven of the patients had small AAAs (mean diameter 37.8 mm), whereas the other 30 patients had large AAAs (mean diameter 54.6mm; Fig 10A and Table 1). In small AAAs, the medial elastic fibers were relatively well preserved in comparison with large AAAs. In large AAAs, the medial elastic laminae were extensively disrupted, and there was a thick ILT (Fig 10B, upper). Nuclear and cytoplasmic expression of HIF-1α was observed in the media and adventitia in both small and large AAAs (Fig 10B, lower). Medial SMCs were positively stained for HIF-1α, which was confirmed with double immunohistological staining with the SMC marker ASMA (Fig 10C). Adventitial fibroblasts were also positively stained for HIF-1α, which was confirmed with double immunohistological staining with the fibroblast marker S-100 (Fig 10D).

The luminal area of the adventitial VV was divided by the total area as bound by the EEL; this showed that they were significantly stenotic in both small and large AAAs in comparison with normal aorta samples from a cadaver, with no significant difference based on size (Fig 10E and 10F).

**Fig 11:**
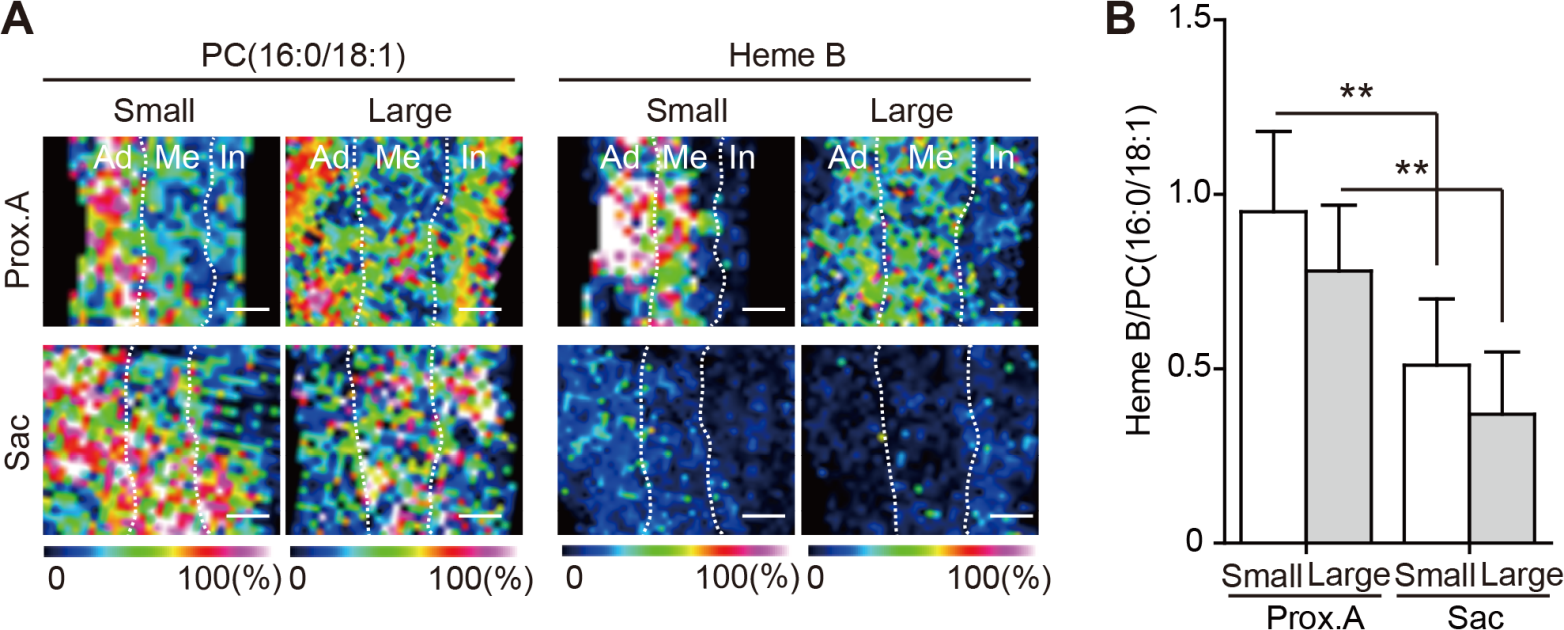
(A) Distribution of phosphatidylcholine (PC)(16:0/18:1) and Heme B analyzed by 500-μm matrix-assisted laser desorption/ionization imaging mass spectrometry (MALDI-IMS). Ad, adventitia; Me, media; In, intima. Scale bar = 500 μm. PC (16:0/18:1) is distributed ubiquitously in each wall layers of both the proximal aorta (Prox.A) and the aneurysmal sac in both sizes of AAA. On the other hand, Heme B was markedly lower in the outer media and the adventitia in the aneurysmal sac than in the proximal aorta. (B) Comparison of the intensity of Heme B relative to PC (16:0/18:1) in the AAA wall between the proximal aorta and aneurysmal sac. **P < 0.01 (a statistically significant difference). Results are means ± standard deviation of 12 independent experiments.

The distributions of both PC(16:0/18:1) and Heme B in the proximal infrarenal aorta and the aneurysmal sac were analyzed by MALDI-IMS. In both large and small AAAs, the Heme B signal was decreased in the AAA sac in comparison with the proximal infrarenal aorta (Fig 11A and 11B)

## Discussion

No drugs have been approved for clinical use in the treatment of AAA, so open-repair with a vascular prosthesis or endovascular repair with a stent-graft are currently the only treatment options [21, 22]. A more detailed and precise understanding of the pathophysiology of AAA will be critical for the development of pharmacological treatment.

Normally, aortic tissue is provided oxygen either through direct diffusion from the aortic blood flow or perfusion via the adventitial VV [23]. The VVs penetrate into the media from the adventitia and form vascular networks. For the ascending aorta, the VVs originate from the coronary ostia and the terminal ventricular branches of the left coronary artery. For the aortic arch, VVs originate from the great vessels of the neck and their proximal branches. On the other hand, VVs of the descending aorta originate from intercostal arteries, and that of the abdominal aorta originate from the lumbar and mesenteric arteries [24]. Adventitial VV blood flow into the abdominal aortic wall can come from three directions: (1) blood flow from the proximal direction via the aortic wall, (2) blood flow from the distal direction via the aortic wall, and (3) blood flow from the perivascular tissues (S1 Fig). In this study, we created rat models with reduced VV blood flow. For that purpose, we blocked the blood flow from the proximal direction via the aortic wall by inserting a polyurethane catheter inside the aorta with a tight ligation over the catheter. The VV blood flow via the proximal aortic wall can be blocked only if the suture ligation was tightly performed over the catheter. Without the ligation, no aneurysmal development occurred. Exfoliation of the aorta also blocked the blood flow from the perivascular tissue and did not cause an aneurysm.

Among the four groups of rats, only the rats in group IV (exfoliation + catheter insertion and a ligation [blockade of the blood flow via the proximal aortic wall]) showed decreased levels of Heme B in the aortic tissue between the ligation and the aortic bifurcation at 24 h after the operation, indicating that hypoperfusion of adventitial VV occurred in the region. Furthermore, the reduction of tissue levels of ATP as well as positive HIF-1α expression in the region suggested perturbation of tissue oxygen metabolism. MALDI-IMS demonstrated that the reduction of both Heme B and ATP distribution were more distinct in the outer layer of the aortic wall (i.e., from the media to the adventitia). Considering that the VV supplied blood flow from the adventitia to the media, the aortic ligation effectively blocked the adventitial VV flow. The placement of the polyurethane tube inside the aorta prevented aortic stenosis by the ligation and maintained luminal blood flow. Both aortic Heme B and ATP depression were consistent at POD 28, suggesting that chronic and sustained hypoperfusion were sustained in group IV. To dismiss concerns that the AAA development might be due to the post-stenotic dilatation, we purposefully created the aortic stenosis using a narrower tube inserted into the aorta (about 50% stenosis) and investigated whether post-stenotic dilatation could develop AAA in the group (S2A–S2C Fig); we could not observe any aneurysmal formation until POD 28 (S2D and S2E Fig). More severe aortic stenosis caused severe bilateral ischemia in the legs due to the aortic occlusion, and the rats died within a few days.

AAA development was observed only in group IV. Therefore, chronic and sustained hypoperfusion can develop AAA. The biological features in the developed AAAs revealed the following: (1) formation of a true aneurysm located infra-renally and fusiform in shape, (2) loss of SMCs and increased degradation of elastin, (3) up-regulated expression and activity of MMP-2 and MMP-9, (4) increased presence of activated macrophages, and (5) the presence of an ILT. Similar findings were reported in other animal models of AAA, such as the elastase-induced AAA model [25, 26] and the calcium chloride-induced AAA models [27, 28], and these biological features are compatible with those in human AAAs [29].

**Fig 12:**
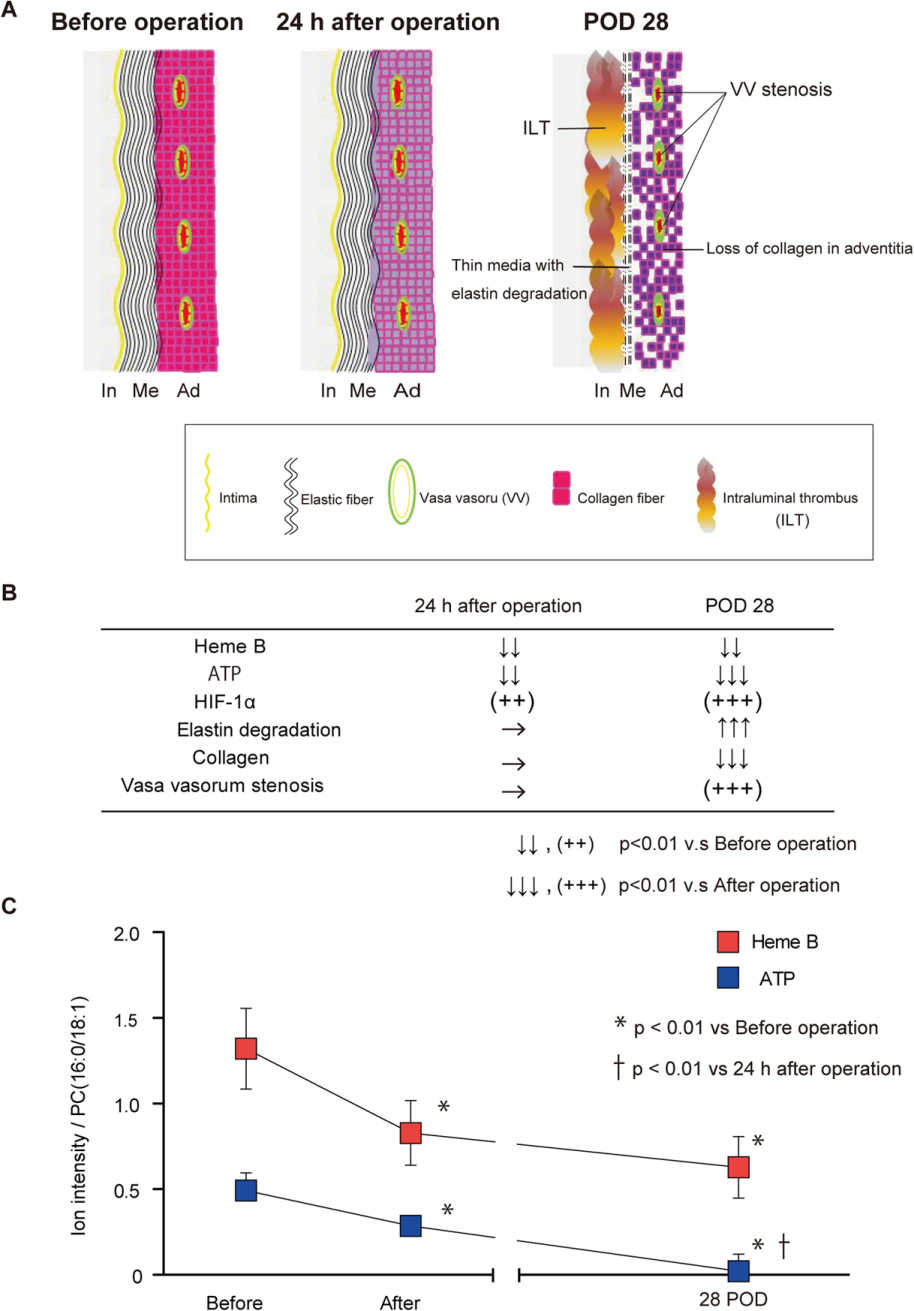
(A) Schematic illustrations of the changes in the abdominal aortae in group IV rats. Before the operation, the aorta consisted of three layers, namely the intima, media, and adventitia, which receive blood flow via the adventitial vasa vasorum (VV). At 24 h after the operation, the three layers of the aorta were kept intact, although VV hypoperfusion and tissue adenosine triphosphate (ATP) depression occurred. At postoperative day (POD) 28, an intraluminal thrombus (ILT) was present. The medial layer became thin with elastin degradation. The adventitia showed loss of collagen and VV stenosis. (B) Summary of the changes in tissue levels of Heme B and ATP, expression of HIF-1α, the amount of elastin degradation, the amount of collagen, and VV stenosis in the aorta at 24 h after the operation and on POD 28. (C) Changes in the tissue levels of Heme B and ATP in the aorta at 24 h after the operation and on POD 28. The intensities of Heme B or ATP relative to phosphatidylcholine (PC;16:0/18:1) with matrix-assisted laser desorption/ionization imaging mass spectrometry were used for the comparison. *P < 0.01 vs. before operation, ✝ P< 0.01 vs. 24 h after the operation.

At POD 28, the AAA in rats in group IV showed a thin medial layer, decreased collagen fiber in the adventitia, and stenosis of the adventitial VV (Fig 12A and 12B). Tissue levels of both Heme B and ATP further decreased at POD 28 compared with those 24 h after the operation, although the depression of Heme B levels was not statistically significant (Fig 12C). Luminal diffusion of oxygen may have been compromised due to the ILT [5]. ILTs are thought to form in the AAA sac during the process of aortic dilatation as a result of platelet accumulation above the aortic bifurcation due to vortex flow and low shear stress [30]. In our models, the ILT was observed at POD 14 when the infrarenal abdominal aorta had already dilated.

At POD 28, the adventitial VVs were found to be stenotic. Previous studies have reported that hypoperfusion of blood vessels might be associated with proliferation of SMCs due to low wall shear stress [31, 32]. In this study, the aortic ligation caused hypoperfusion of the VV, which may have induced SMC proliferation, leading to VV stenosis. The resultant VV stenosis might further decrease the perfusion, which might exacerbate tissue hypoxia in the aneurysmal sac. Indeed, liquid chromatography-tandem mass spectrometry demonstrated significant depression of tissue levels of ATP, ADP, NADP, and NADPH in the aneurysmal sac, indicating a disturbance of aerobic metabolism.

The histological study of human AAA samples revealed that adventitial VVs were also stenotic as a result of proliferation of SMCs, not only in large AAAs but also in small AAAs, suggesting that arteriosclerosis of the VV may occur early in AAA development. Considering that both cigarette smoking and hypertension are major risk factors for AAA [2], it is possible that AAA may develope as a result of arteriosclerotic changes in the VV and its hypoperfusion.

In the human AAA samples, MALDI-IMS similarly demonstrated that the tissue levels of Heme B were significantly lower in the sac than in the proximal infrarenal aorta in both small and large AAAs. HIF-1α was also positively stained in human AAAs, indicating that hypoxia may occur not only as a terminal phenomenon in an enlarged AAA but at the beginning of AAA development.

With regard to the distribution of the adventitial VV, the infrarenal abdominal aorta has inherently fewer VV than dose the thoracic aorta [23], so the infrarenal aorta is particularly susceptible to hypoxia. Indeed, more than 94% of AAAs occur in the infrarenal aorta[33], which may account for the difference in pathogenesis between thoracic aortic aneurysms and AAAs [34]. Thoracic aortic aneurysms is mainly genetic in origin, whereas AAA is a multifactorial condition with an arteriosclerotic background [35].

Chronic hypoxia in the aortic tissue may trigger biochemical responses that augment inflammation, causing loss of tissue integrity; these responses may include (1) increased secretion of MMP-2 [8]; (2) appearance of macrophages in the intima and media [7]; and (3) impaired synthesis of elastin and collagen[36, 37], which were also observed in our models. The gradual aneurysm development in our model suggests that chronic and sustained hypoxia is associated with aneurysm development in the infrarenal abdominal aorta.

In conclusion, our novel rat model, in which chronic hypoxia due to hypoperfusion of the adventitial VV was created by polyurethane catheter-insertion and a suture ligation of the aorta, developed aneurysms with characteristics similar to those of human AAAs. In human AAAs, adventitial VVs were stenotic and the aortic wall was ischemic in both small and large aneurysms. These results indicate that chronic VV hypoperfusion can develop infrarenal AAA. Thus, inhibition of adventitial VV stenosis and maintenance of perfusion prior to aortic aneurysm formation could be targets for pharmacological intervention.

## Supporting Information

S1 Fig Schematic of adventitial vasa vasorum (VV) blood flow into the abdominal aortic wall. Adventitial VV blood flow into the abdominal aortic wall can be classified to three types: (1) blood flow from the proximal direction via the aortic wall, (2) blood flow from the distal direction via the aortic wall, and (3) blood flow via the perivascular tissues.
(TIF)

S2 Fig Schematics of a rat model of aortic stenosis. (n = 10). (A)(B) Each step of the procedure induce abdominal aortic stenosis is shown. The operation consisted of the following steps. (1) The infra-renal aorta was exfoliated from the surrounding tissue. (2) A thin polyurethane catheter was placed longitudinally along the ventral surface of the aorta and a 4–0 silk suture was tied around both the aorta and the catheter as depicted in B-1,1’. Scale bar = 5 mm (3) The catheter is then removed. (4) Blood flow restarted with aortic stenosis as shown in B-2,2’. (scale bar = 5 mm). (**C)**. External diameter of the thin catheter was 50% compared to that of the catheter inserted into the aorta in Fig 1B. (**D**) Maximum aortic diameters measured with transabdominal ultrasonography. The aortic diameter of the post-stenotic aorta was not increased in this model. The data of the aortic diameters are shown as means ± standard deviations. Comparisons were made using analysis of variance followed by Tukey’s post-test. (**E**) Incidence of abdominal aortic aneurysm (AAA) in rats. An aneurysm was defined as a more than 50% increase in the aortic diameter over baseline level. Data were analyzed using a Kaplan–Meier analysis.
(TIF)
