# Supplementary figures and images for: Hypoperfusion of the Adventitial Vasa Vasorum Develops an Abdominal Aortic Aneurysm
Source: PLoS One. 2015 Aug 26;10(8):e0134386. doi: 10.1371/journal.pone.0134386 (PMC4550325; doi:10.1371/journal.pone.0134386)

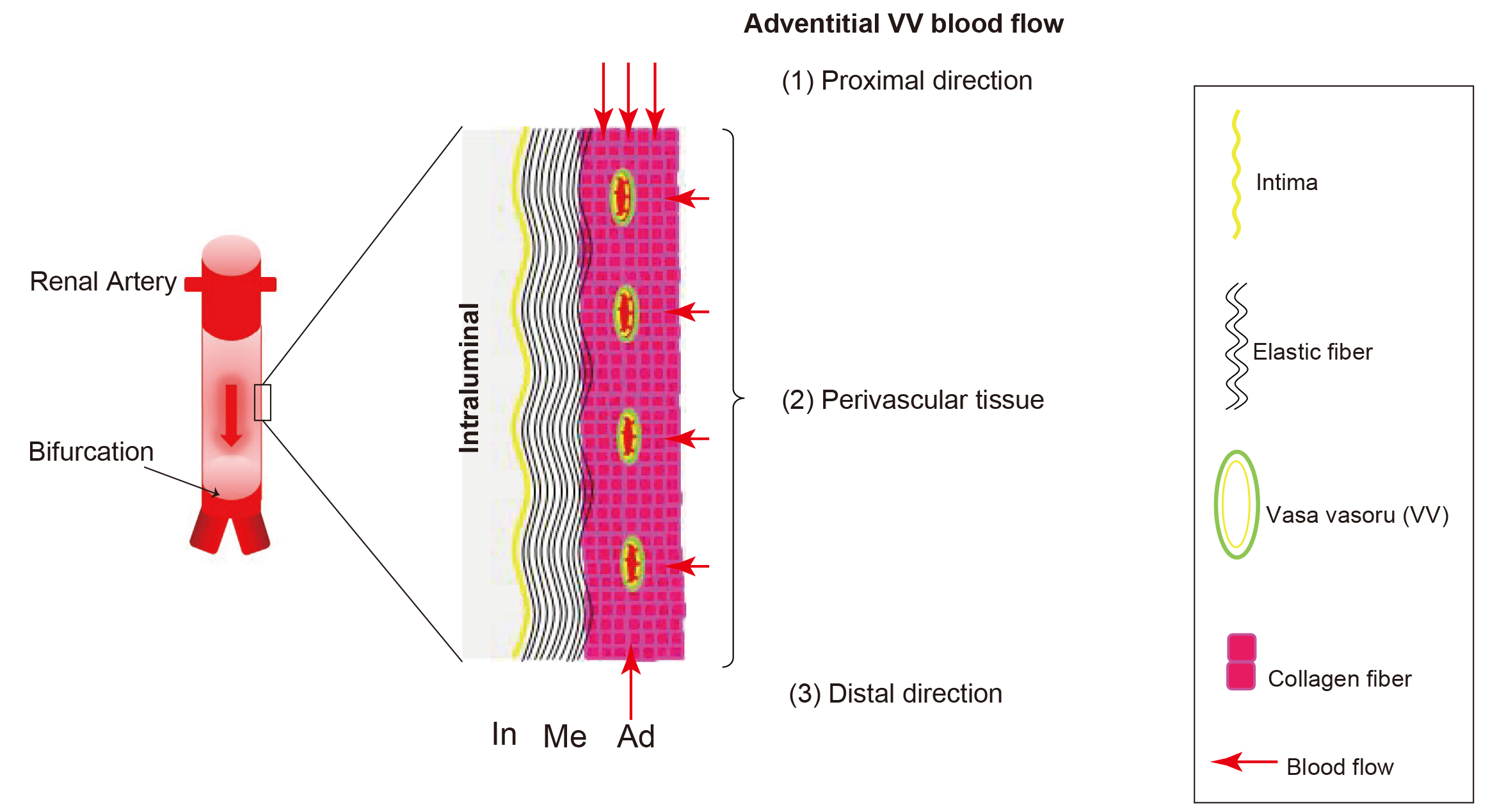

Supplement: S1 Fig — Adventitial VV blood flow into the abdominal aortic wall can be classified to three types: (1) blood flow from the proximal direction via the aortic wall, (2) blood flow from the distal direction via the aortic wall, and (3) blood flow via the perivascular tissues. (TIF) [file pone.0134386.s001.tif]

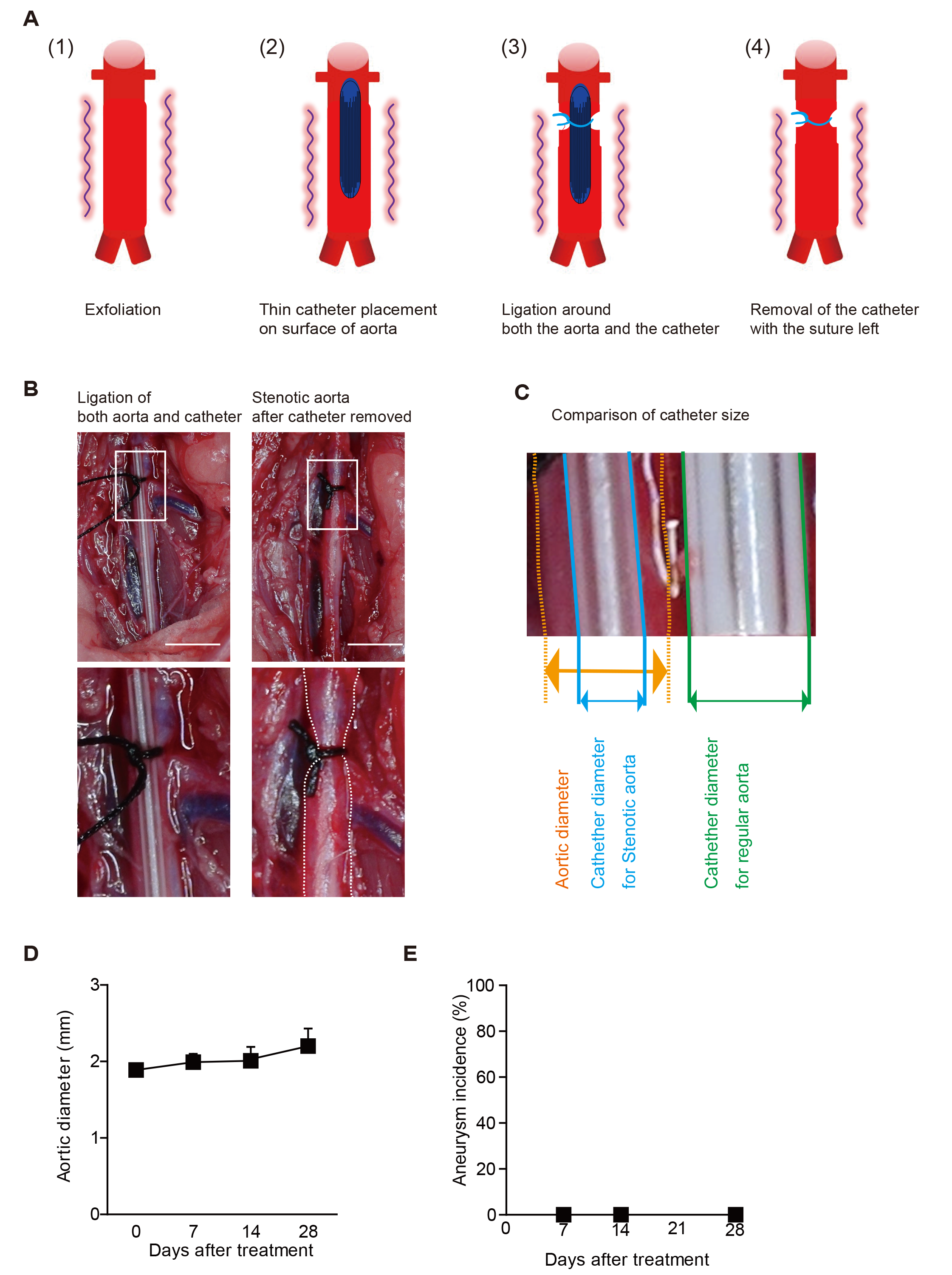

Supplement: S2 Fig — (A)(B) Each step of the procedure induce abdominal aortic stenosis is shown. The operation consisted of the following steps. (1) The infra-renal aorta was exfoliated from the surrounding tissue. (2) A thin polyurethane catheter was placed longitudinally along the ventral surface of the aorta and a 4–0 silk suture was tied around both the aorta and the catheter as depicted in B-1,1’. Scale bar = 5 mm (3) The catheter is then removed. (4) Blood flow restarted with aortic stenosis as shown in B-2,2’. (scale bar = 5 mm). (C). External diameter of the thin catheter was 50% compared to that of the catheter inserted into the aorta in Fig 1B. (D) Maximum aortic diameters measured with transabdominal ultrasonography. The aortic diameter of the post-stenotic aorta was not increased in this model. The data of the aortic diameters are shown as means ± standard deviations. Comparisons were made using analysis of variance followed by Tukey’s post-test. (E) Incidence of abdominal aortic aneurysm (AAA) in rats. An aneurysm was defined as a more than 50% increase in the aortic diameter over baseline level. Data were analyzed using a Kaplan–Meier analysis. (TIF) [file pone.0134386.s002.tif]
